# Clinical Outcomes of Patients With Acute Myocardial Infarction in Health Professional Shortage Areas in Indiana

**DOI:** 10.1016/j.jacadv.2024.100906

**Published:** 2024-03-08

**Authors:** David J. Gunderman, Ashish Kumar, Raymundo Munguia-Vazquez, Keyur Vora, Chirag Shah, Nathan Lambert, Brendan Cavanaugh, Rohan Dharmakumar, Ankur Kalra

**Affiliations:** a Indiana University School of Medicine-West Lafayette, West Lafayette, Indiana, USA; b Cleveland Clinic Akron General, Akron, Ohio, USA; c Krannert Cardiovascular Research Center, Indiana University School of Medicine, Indianapolis, Indiana, USA; d IU Health Physicians Cardiology, Fishers, Indiana, USA; e IU Health Arnett, Lafayette, Indiana, USA; f Franciscan Health, Lafayette, Indiana, USA

Health professional shortage areas (HPSAs) are defined by the Federal Health Resources and Service Administration and are used by federal programs to incentivize physicians to provide care in shortage regions.^1^ A significant barrier in previous research has been the complexity of accurately correlating deidentified patient addresses, typically limited to zip codes, with HPSA status;^2^ these studies typically classify patients in HPSAs only if their entire zip code lies within such areas. Recently, a novel, more precise method was validated for correlating zip codes with Housing and Urban Development census tract regions, which are used to define HPSAs.^3^ This study applies this method to address a critical gap in understanding the impact of HPSA status on individual patient outcomes in acute myocardial infarction (AMI), where timely access to treatment is crucial.^4^ The objective of this study was to determine if HPSA status is associated with AMI risk factors, comorbidities, medical treatment, laboratory values, and clinical outcomes at the time of presentation.

We used the Department of Housing and Urban Development’s crosswalk file,^5^ which links zip codes to census tracts, to calculate the proportion of residences within each zip code situated within each HPSA-designated census tract; the weighted average of these proportions represents the proportion of residences, and by extension, patients within a specific zip code that lie within a HPSA.^3^

Patients who presented with AMI (ie, presenting with ST-segment elevation myocardial infarction and who received percutaneous coronary intervention [PCI]) at 7 hospitals encompassing both rural and urban Indiana between 2009 and 2022 were categorized into HPSA and non-HPSA groups using a HPSA residence proportion cutoff of 50%. Patients in zip codes with more than 50% of residences in HPSAs were classified as living in a HPSA, and those in zip codes with 50% or less were classified as non-HPSA. We also performed a sensitivity analysis to determine if results changed when using the continuous residence proportion HPSA variable vs the binary HPSA/non-HPSA variable with a 50% cutoff. Data were extracted from the local National Cardiovascular Data Registry, which includes baseline characteristics. Adverse clinical outcome data were extracted by querying the IU Health electronic medical record data. We used t-tests for continuous variables, chi-square tests for categorical variables with expected counts of at least 5, and Fisher’s exact tests for categorical variables with expected counts of <5 to compare the 2 patient groups by sex, self-reported race/ethnicity, age, AMI risk factors, comorbidities, medications, laboratory values, and adverse clinical outcomes.

We used multivariable logistic regression models to determine the independent association between HPSA status and each adverse clinical outcome variable. Adverse clinical outcome variables all occurred during the same encounter as the AMI and included in-hospital mortality, post-PCI bleed, cerebrovascular accident, and an outcome variable that included all 3 of the other adverse clinical outcomes, which we termed adverse events. We included as covariates in each model all variables with more than 90% complete data, including age, race/ethnicity, gender, hypertension, diabetes, dyslipidemia, prior myocardial infarction, prior PCI, cerebrovascular disease, chronic lung disease, prior coronary artery bypass grafting, dialysis, peripheral arterial disease, and body mass index.

Age is summarized using median (range). Categorical variables are summarized using count (%). Numerical variables are summarized using mean (95% CI). A level of significance of 0.05 was used for all hypothesis testing.

Out of the 8,348 patient records obtained, 5,911 had recorded zip codes. Among these, 2,473 patients were in the 213 zip codes with at least 50% of residences within HPSAs, and 3,438 were in the 353 zip codes with <50% of residences within HPSAs. A total of 4,179 (70.7%) were male, 5,183 (87.7%) were White, 411 (7.0%) were Black/African American, and the median age was 60 (range: 24-99). Our analysis revealed no significant association between HPSA status and adverse clinical outcomes in AMI patients, even when adjusting for potential confounders (Table 1). Certain demographic and clinical factors, including systolic blood pressure, race/ethnicity, tobacco use, and P2Y12 medication prescription distribution were found to differ between the HPSA and non-HPSA groups (Table 1). Our sensitivity analysis revealed no differences in results when using the continuous HPSA variable vs the binary HPSA variable.

**Table 1:** Comparative Analysis of Baseline Demographics and Clinical Outcomes After Myocardial Infarction in HPSA vs Non-HPSA Regions

	Non-HPSA	HPSA	Univariable *P* Value
Total	3,438 (58.2%)	2,473 (41.8%)	
Demographics			
Male	2,310 (70.1%)	1,869 (71.5%)	0.25
Race and ethnicity			<0.001^a^
Hispanic or Latino	47 (1.4%)	30 (1.1%)	
Multiple	66 (2%)	58 (2.2%)	
American Indian/Alaskan Native	9 (0.3%)	3 (0.1%)	
Asian	36 (1.1%)	12 (0.5%)	
Black/African American	285 (8.6%)	126 (4.8%)	
Hawaiian/Pacific Islander	6 (0.2%)	0	
White	2,816 (85.4%)	2,367 (90.5%)	
Unknown	31 (1.0%)	19 (0.7%)	
Age	60 (24-99)	60 (24-97)	0.33
Comorbidities/risk factors			
Cerebrovascular disease	246 (7.5%)	188 (7.2%)	0.73
Chronic lung disease	382 (11.6%)	337 (12.9%)	0.14
Dialysis	26 (0.8%)	23 (0.9%)	0.77
Peripheral arterial disease	227 (6.9%)	198 (7.6%)	0.31
Hypertension	2,194 (66.6%)	1,711 (65.4%)	0.36
Diabetes	928 (28.2%)	786 (30.1%)	0.11
Dyslipidemia	1,980 (60.1%)	1,508 (57.7%)	0.06
Tobacco use			0.002^b^
Current	614 (42.7%)	580 (48.9%)	
Former	317 (22.1%)	258 (21.7%)	
Never	506 (35.2%)	349 (29.4%)	
Prior heart failure	77 (4.3%)	43 (3.1%)	0.09
Prior valve surgery	5 (0.3%)	6 (0.4%)	0.55
Prior CABG	127 (3.9%)	104 (4%)	0.84
Prior MI	665 (20.2%)	514 (19.7%)	0.62
Prior PCI	748 (22.7%)	552 (21.1%)	0.15
Diagnostics			
Creatinine, mg/dL	1.14 (1.12-1.17)	1.11 (1.08-1.14)	0.12
BMI, kg/m^2^	30.4 (30.1-30.6)	30.2 (29.9-30.5)	0.41
Total cholesterol, mg/dL	173 (162-184)	171 (155-187)	0.88
HDL, mg/dL	37 (34-39)	40 (37-43.6)	0.1
Heart rate, beats/min	94 (83-100)	91 (81-100)	0.67
Systolic BP, mm Hg	143 (142-145)	140 (139-142)	0.01^c^
LVEF	54 (52-56)	51 (49-53.8)	0.08
Medications			
ACE inhibitor	1,667 (57.1%)	1,378 (57.6%)	0.72
Antiplatelet/aspirin	2,947 (98.1%)	2,371 (97.7%)	0.25
ARB	404 (13.5%)	348 (14.4%)	0.34
Beta-blocker	2,746 (95.1%)	2,222 (94.8%)	0.66
Statin	2,971 (98.9%)	2,396 (98.7%)	0.54
Nonstatin	193 (6.4%)	134 (5.5%)	0.17
P2Y12, clopidogrel	941 (31.3%)	985 (40.4%)	<0.001^a^
P2Y12, prasugrel	573 (19.1%)	288 (11.8%)	<0.001^a^
P2Y12, ticagrelor	1,503 (54.8%)	1,158 (50.4%)	0.002^b^
Anticoagulant, warfarin	16 (1.2%)	22 (1.9%)	0.19
Apixaban	94 (7.1%)	76 (6.7%)	0.69
Rivaroxaban	26 (2%)	14 (1.2%)	0.2
Long-acting nitrate	129 (16.7%)	110 (19.1%)	0.28

	Outcomes				
	Non-HPSA	HPSA	Univariable *P* Value	Adjusted OR (95% CI)	Multivariable *P* Value
Encounter death	108 (3.3%)	88 (3.4%)	0.88	1.03 (0.77-1.37)	0.85
Cerebrovascular accident	46 (1.4%)	46 (1.8%)	0.29	1.27 (0.84-1.91)	0.26
Bleed	43 (1.3%)	25 (1%)	0.22	0.73 (0.44-1.20)	0.21
Adverse event	196 (5.9%)	158 (6%)	0.91	1.02 (0.82-1.26)	0.88

Values are n (%) or mean (range) unless otherwise indicated. Significance level: ^a^0.001, ^b^0.01, ^c^0.05.

ACE = angiotensin-converting enzyme; ARB = angiotensin receptor blocker; BMI = body mass index; BP = blood pressure; CABG = coronary artery bypass graft; HDL = high-density lipoprotein; HPSA = health professional shortage area; LVEF = left ventricular ejection fraction; MI = myocardial infarction; PCI = percutaneous coronary intervention.

In this study, we applied a novel method for estimating HPSA status based on patient zip codes, providing a valuable tool for geographic health care disparity research. Limitations of this study include potential inaccuracies from using zip codes to determine HPSA status, missing data, limited generalizability due to inclusion of a single health system, lack of inclusion of potentially important confounders (eg, insurance status, income level, adequate preventative care), and potential bias from its retrospective design. Analyzing AMI patients in Indiana, we found significant differences in demographic and clinical characteristics between those in HPSA and non-HPSA areas, including systolic blood pressure, race/ethnicity, tobacco use, and P2Y12 medication prescription distribution. However, HPSA status did not significantly impact adverse clinical outcomes in AMI patients, even after adjusting for potential confounding factors. Our findings highlight the need for a nuanced understanding of the factors influencing patient health in HPSA-designated regions. Future work could include collaborating with policy experts to translate these insights into actionable changes, such as developing targeted interventions for improving access and outcomes in HPSA-designated areas.
